# Retracted: Clinical Efficacy and Safety Analysis of PD-1/PD-L1 Inhibitor vs. Chemotherapy in the Treatment of Advanced Non-Small-Cell Lung Cancer: A Systematic Review and Meta-Analysis

**DOI:** 10.1155/2022/9808495

**Published:** 2022-12-25

**Authors:** BioMed Research International

*BioMed Research International* has retracted the article titled “Clinical Efficacy and Safety Analysis of PD-1/PD-L1 Inhibitor vs. Chemotherapy in the Treatment of Advanced Non-Small-Cell Lung Cancer: A Systematic Review and Meta-Analysis” [1] due to concerns that the peer review process has been compromised.

Following an investigation conducted by the Hindawi Research Integrity team [2], significant concerns were identified with the peer reviewers assigned to this article; the investigation has concluded that the peer review process was compromised. We therefore can no longer trust the peer review process and the article is being retracted with the agreement of the editorial board.

The authors do not agree to the retraction.
